# Invitation Choice Structure Has No Impact on Attendance in a Female Business Training Program in Kenya

**DOI:** 10.1371/journal.pone.0109873

**Published:** 2014-10-09

**Authors:** Faizan Diwan, Grace Makana, David McKenzie, Silvia Paruzzolo

**Affiliations:** 1 Innovations for Poverty Action, Kisumu and Kakamega, Kenya; 2 Development Research Group, World Bank, Washington, DC, United States of America; 3 International Labour Organization, Geneva, Switzerland; Baylor College of Medicine, United States of America

## Abstract

Business training programs are a common form of support to small businesses, but organizations providing this training often struggle to get business owners to attend. We evaluate the role of invitation choice structure in determining agreement to participate and actual attendance. A field experiment randomly assigned female small business owners in Kenya (N = 1172) to one of three invitation types: a standard opt-in invitation; an active choice invitation where business owners had to explicitly say yes or no to the invitation; and an enhanced active choice invitation which highlighted the costs of saying no. We find no statistically significant effect of these alternative choice structures on willingness to participate in training, attending at least one day, and completing the course. The 95 percent confidence interval for the active treatment effect on attendance is [−1.9%, +9.5%], while for the enhanced active choice treatment it is [−4.1%, +7.7%]. The effect sizes consistent with our data are smaller than impacts measured in health and retirement savings studies in the United States. We examine several potential explanations for the lack of effect in a developing country setting. We find evidence consistent with two potential reasons being limited decision-making power amongst some women, and lower levels of cognition making the enhanced active choice wording less effective.

## Introduction

Business training is one of the most common forms of active support provided by governments, development agencies, and NGOs throughout the world. However, a common problem facing organizations that offer this training is how to get small business owners to attend. A recent summary of business training programs in developing countries found that even when training is offered for free, along with travel or food supplements, on average only 65 percent of those who were invited to attend did so [1]. Low attendance can occur even when dealing with individuals who had initially expressed interest in attending such a course, with studies in Bosnia-Herzogovina and Peru reporting take-up rates of 39 percent and 51 percent respectively, despite having screened for initial interest in training [2], [3]. This would not be as much of a concern if those who choose not to attend are doing so because they would not benefit from training, but there are at least two studies which suggest that individuals who are initially the least interested have the most to gain from business training [4], [5].

This problem of incomplete take-up is not unique to business training programs, but instead is a common feature of a wide range of public policy interventions around the world. A recent literature has emphasized the importance of the choice architecture, or context in which decisions are presented and made, in determining program participation [6], [7]. One of the best known policies that arise from this has been the use of “opt-out” defaults, in which people are automatically enrolled in some program unless they explicitly choose otherwise. Such policies have worked well to increase take-up in situations where a one-shot decision is all that is required, such as signing up to be an organ donor or to allow automatic deductions from a worker's pay to their retirement savings account. But there is a concern that “because opt-out policies yield decisions through the inaction of the decision maker, they are less likely to engender the kind of committed follow-up that is often useful when it comes to implementing the decision ([8], p.377).

An alternative approach that has been proposed has been to require individuals to make an active choice, explicating choosing between various options rather than being defaulted to any particular option. This approach led to a 28 percentage point increase in participation in a 401k retirement savings program compared to an opt-in policy where employees had to choose to enroll [9], and to be 20 percentage points more likely to want a flu shot in a lab experiment [8]. Enhanced active choice provides a refinement of the active choice approach, in which the respondent again has to actively choose between competing options, but with choices presented in a way to highlight the losses involved in choosing the policy option not preferred by the policymaker. The use of enhanced active choice led to 9.6 to 16 percentage point increases in a prescription refill program offered by a major drugstore compared to an opt-in policy [8].

Behavioral theory offers several reasons why active choice and enhanced active choice structures can improve on a standard policy of requiring individuals to opt-in [7], [8]. Active choosing can overcome inertia and procrastination, forcing individuals to incur the effort costs involved in deciding between options that might otherwise have not been incurred. Moreover, there is evidence to suggest that the very act of making a decision can increase the decision-makers satisfaction with this decision [10], and their commitment to following through with it [11] compared to the case of passive decision-making. This suggests that the approach could be useful in situations like attempting to get small business owners to show up to business training, in which the initial decision of whether or not to attend needs to be followed up by their own actions of subsequently travelling to the training sessions.

Our study uses a randomized experiment with female small business owners in Kenya to test the effectiveness of different choice structures in determining the decision to attend business training and the ultimate attendance rates in this training. This represents the first application of this method in a developing country setting. We find that active choice and enhanced active choice invitations have no significant impact on getting individuals to agree to attend training, or to actually attend, compared to a standard opt-in policy. This is despite large differences in training attendance rates according to observable characteristics of the women and their businesses. We speculate that, and find evidence consistent with, a reason for the lack of effect being that decision-making power does not always rest with the women being invited, and that lower education levels may limit the effectiveness of more complicated enhanced choice decision structures.

## Materials and Methods

The target population for our study consists of female microenterprise owners in Kenya selected to be invited to participate in the International Labor Organization (ILO) 's GET (Gender and Entrepreneurship Together) Ahead business training program as part of a randomized controlled trial designed to measure the impact of this program. Human Subjects Approval was obtained from Innovations for Poverty Action (13February-002) and the Maseno University Ethics Review Committee (MSU/DRPC/MUERC/000006/13). Authority to conduct research in Kenya was provided by the Kenyan Ministry of Science and Technology (NCST/RCD/14/013/553B). Written informed consent was obtained from participants, with two copies of the form signed, one kept by the participant, and one kept by the survey organization (Innovations for Poverty Action). Those individuals with low education who could not sign their names were given the option of using a thumbprint in place of a signature. Again two copies of the form were signed, one for the participants and one kept with the survey organization. These procedures were approved by both ethics committees.

### Selecting the study population

Four of Kenya's 47 counties were chosen as the locations for the study: Kakamega and Kisii in the Western region, and Embu and Kitui in the Eastern region. This choice was made based on a number of factors, including the desire for geographic variation, the fact that these counties had not previously been involved in ILO training, and consultation with various stakeholders including training providers, women's enterprise development groups, and government. These four regions are largely rural, with populations between 500,000 and 1.6 million, and the majority of the population below the poverty line in all regions except Embu. We do not have data on business owners in other Kenyan counties to compare to our owners, but do not believe there are likely to be large differences with businesses in other rural Kenyan counties.

In each of the four counties, field staff from Innovations for Poverty Action, Kenya, mapped out all market centers deemed as medium or large outside of the main cities. Field staff then conducted a market census of female-owned enterprises operating on a non-market day in these markets. The listing operation took place one county at a time between June 3, 2013 and November 1, 2013. Altogether 6,296 businesses in 161 markets were listed.

We then applied an eligibility filter to determine which women to include in the baseline survey. This filter required the women to have reported profits, and not to have reported profits that exceeded sales; to have a phone number that could be used to invite them for training; to be 55 years or younger in age; to not be running a business that only dealt with phone cards or m-pesa, or that was a school; that the person responding not be an employee; that the business not have more than 3 employees; that the business have profits in the past week between 0 and 4000 Kenyan Shillings (KSH) (1 US Dollar averaged approximately 85 KSH over the survey period); that sales in the past week be less than or equal to 50,000 KSH; and that the individual had at least one year of schooling. These criteria were chosen to reduce the amount of heterogeneity in the sample (thereby increasing our ability to detect treatment effects), and to increase the odds of being able to contact and find individuals again. Applying this eligibility filter reduced the 6,296 individuals to 4,037 individuals (64%).

Baseline surveys took place soon after the listing surveys in each county, between June and November 2013. Out of a target of 4,037 individuals, we were able to interview 3,537 (87.6%) in time to consider them for inviting to training. The main reasons individuals were not interviewed were that they were travelling away from their business, along with some refusals.

This left us with a sample of 3,537 individuals in 157 markets who had satisfied the screening criteria and completed the baseline survey. The Baseline survey data and questionnaire are available in the World Bank's Open Data Library http://microdata.worldbank.org/index.php/catalog/1985. The Stata do file to replicate the tables in this paper is available at https://drive.google.com/file/d/0B9C9RwWKZrUNLWRmNzJZdFlQeFU/edit?usp=sharing and will also be put in the World Bank open data library.

Markets were then stratified by geographic region and by the number of women interviewed in the market, and 93 of the 157 markets were randomly selected to have individuals in them invited for training. Within each market, individuals were assigned to be invited to training, or to the control condition for the main study (not be invited to training) within markets by forming four strata, based on quartiles of weekly profits from the market census (< = 450 KSH, 451–800 KSH, 801–1500KSH, 1501–4000KSH), and then assigning half the individuals within each strata to training. When the number of individuals in the strata was odd, the odd unit was also randomly assigned to training. This resulted in 1172 of the 2160 individuals in selected markets being assigned to be invited to training. This forms the sample to be used in this paper's experiment.

### Training Course

The training that was being offered is the ILO's Gender and Entrepreneurship Together – GET Ahead for Women in Enterprise program. This program highlights essential entrepreneurial skills from a gender perspective, addressing the practical and strategic needs of low-income women in enterprise by strengthening their basic business and people management skills. It shows women how to develop their personal entrepreneurial traits and obtain support through groups, networks and institutions dealing with enterprise development” [12]. The program began in Thailand in 2001, and over the next decade was used in 18 countries around the developing world [13].

An objective of the program is to create a “business mind” among low-income women engaged in small-scale businesses. The training methodology is participatory, with practical exercises to teach concepts. Topics covered included several gender concepts that tend not be emphasized in general business training programs such as: the difference between sex and gender, and the role of cultural constraints in shaping women in business; dividing household and business tasks; and how to network with other women and the role of women's associations. In addition, it covers a number of topics more typical of standard programs such as recordkeeping and bookkeeping; separating business and household finances; marketing; financial concepts; costing and pricing; generating and fine-tuning new business ideas; setting smart objectives; and traits needed for business success. The materials are designed to be used with low-income women, and use participatory and practical training methods. As a result, the providers of the course believe it is suitable even for women with very low literacy levels (recall we screen out women with no schooling, and only 8.8 percent of our sample has fewer than 6 years of schooling).

The workshop lasts 5 days, and was offered in two to three locations per county. The locations were chosen to be relatively central to clusters of marketplaces, and were typically held in local hotels or town buildings. Participants were provided transport subsidies of approximately $6 per day to cover the costs of travelling from their residences to these locations. The median marketplace had a straight-line distance of 14.3 kilometers from the training location, with a 25–75 range of 8.2 to 23.2 kilometers. The course was offered for free to those invited, but the estimated cost of provision per woman trained is between $US222 and $US333.

### Random assignment of invitation type treatments

The 1172 women selected to be invited for training were further randomly assigned to receive one of three different invitation types, with randomization occurring within market. Table 1 shows the geographic breakdown of this assignment.

**Table 1:** Number of Women Allocated to Each Treatment by Region.

		Active	Enhanced
County	Opt-in	Choice	Active Choice
Kakamega	85	87	85
Kisii	92	90	92
Embu	78	75	78
Kitui	138	134	138
**Total**	393	386	393

Individuals received one of three types of invitations, which were read to them in Swahili by IPA project staff. All three invitations explained that the course was a 5 day workshop on business skills, and that the objective of this workshop is to “train you and build your capacity to operate your business or enable you to set up and operate a new business efficiently. This training typically costs 17,000 KSH to provide, and even though it is usually subsidized, most NGOs charge at least 2,000 KSH for it. We are pleased to offer it to you for free.”

In order to attribute any differences in behavior to differences in choice structure, it is essential that all three groups receive the same information. We closely follow the approach used in [8] here – they have a common script for all three invitation types, with the only difference then being how the choice decision is presented following this information. This is also what we do here. The supporting materials provide examples of the script used in Swahili, along with an English translation.

The first invitation type was the standard opt-in approach, in which 393 invited women had to say if they wanted to participate. The second invitation involved an active choice, in which 386 invited women were asked to make an explicit choice between participating and not participating. The third invitation involved an enhanced active choice, in which 393 invited women were asked to make an explicit choice between participating and not participating, after highlighting the benefits involved in participation and cost of not participating. Specifically, the wording used was:

- ***Opt-in***: *please let me know if you will participate in the GET AHEAD training program*
- ***Active Choice***: *Please let me know if*

 
*you will participate in the GET AHEAD training program*
 
*you will not participate in the GET AHEAD training program*

- ***Enhanced Active Choice***: *Please let me know if*

 
*you will participate in the GET AHEAD training program in order to learn new business skills that could help you grow your business and take advantage of free training valued at KSH 17,000*.
 
*you will not participate in the GET AHEAD training program, and will turn down the opportunity to learn new skills for your business and choose not to receive KSH 17,000 of free training*.

Note that the enhanced active choice explicitly highlights the benefits of participating (new skills to help grow the business, free training) and the costs of not participating (losing the opportunity to gain these new skills, and losing training valued at KSH 17,000). Again this follows closely the wording used for the enhanced active choice treatment in [8].

If women were reading the invitations themselves, these questions would be phrased in the first-person (e.g. I will participate…”). However, given a setting where some women have low education levels, and a desire to make sure everyone understands the invitation, these questions had to be phrased in the second person to make sense when being read to them by someone else.

### Verification of Random Assignment and Characteristics of the Sample

Table 2 provides a description of the individual and business characteristics of the women in our study, and verifies that randomization succeeded in providing comparable groups across the three invitation types. The average woman in our study is 36 years old and has almost 9 years of completed schooling. Two-thirds of the women are married, 92 percent have at least one child, and 47 percent have a child aged 5 and under.

**Table 2:** Verification of Randomization for Individual Characteristics Table.

	Opt-in	ActiveChoice	EnhancedActiveChoice	p-value fortest ofequality ofmeans
*Individual Characteristics*				
Age	35.9	36.5	35.5	0.298
Aged above 35	0.481	0.516	0.455	0.299
Years of Schooling	8.81	8.91	9.03	0.542
Married	0.673	0.689	0.662	0.723
Has a child	0.913	0.914	0.926	0.768
Has a child of 5 and under	0.445	0.491	0.471	0.400
Household Size	4.845	4.992	5.077	0.265
High discount rate	0.514	0.518	0.539	0.746
Hyperbolic discounter	0.267	0.267	0.265	0.973
Raven test score	6.857	7.058	6.784	0.239
Digitspan recall	4.926	5.026	4.939	0.303
Owner has previously participated in business training	0.074	0.101	0.066	0.220
*Business Characteristics*				
Sector is retail	0.772	0.770	0.780	0.906
Sector is services	0.228	0.230	0.220	0.906
Weekly hours worked by owner in business	59.295	59.930	60.160	0.772
Age of firm in years	6.753	6.171	6.241	0.389
Keeps business records	0.328	0.373	0.313	0.215
Has an employee who is not a family member	0.135	0.130	0.107	0.433
Profits in last week (KSH)	1142	1128	1115	0.915
Capital stock (excluding land and buildings) (KSH)	30394	29770	26635	0.588
Total business practices score	12.263	12.704	11.655	0.019
Straight line distance to training location (km)	19.671	18.959	19.671	n.a.
Distance to training above 10 km	0.653	0.649	0.653	n.a.

Notes: p-value for test of equality of means controls for randomization at the market level.

n.a. denotes not applicable since there is no variation in this variable within markets.

They work full-time in their businesses, averaging 60 hours per week. 77 percent of these businesses are in the retail sector. The most common activity is selling fruit and vegetables, which approximately one-third of firms do. The next most common business types are selling household goods, dressmaking, selling grains and cereals, and operating a food kiosk or small restaurant.

The businesses are small in size. Average weekly profits are 1128 KSH (US$13), mean capital stock including raw materials and inventories is 28934 KSH (US$340), median capital stock is only 10200 KSH (US$120), and only 12 percent have a non-family member employed in the firm. The cost of providing the training is thus approximately twice the total capital stock of the median firm.

Our baseline survey contains several additional measures of individual and firm characteristics that might be predicted to influence training take-up. Since the costs of training are immediate (time away from their business, the hassle of travelling) and the benefits occur in the future, individuals with high discount rates or time-inconsistent preferences may be less likely to attend. We elicit discount rates via standard hypothetical questions asking them what amount would they be willing to accept today instead of 1000 KSH in one month, and then similarly for five versus six months. Individuals willing to take 800 KSH or less today (the median) are classified as having high discount rates. The scope for improving their business may depend on their previous participation in training, education level, innate ability (measured by a Raven test and digit-span recall test), and level of existing business practices (measured using the index of [14]). Finally, we would expect higher attendance for those with shorter distances to travel, and use the GPS coordinates of the marketplaces and training locations to calculate straight-line distances.

### Outcomes of interest and hypotheses

Our own administrative records provide three measures related to attendance. The first measure is whether or not they say they will attend when invited. The second measure is whether they show up for at least one day of training, and the third is whether they attend all five days of training. Re-invitations were only done on a limited basis reflecting the logistics of organizing the training sessions. 169 of those who did not attend were re-invited to a subsequent training session in their region, of which only 13 individuals attended. We do not use the re-invitees in our attendance measures, since the script for re-invitation was the same for all three treatment groups. Attendance after re-invitation is reasonably balanced across the three invitation types: 5 from the opt-in, 5 from the active choice, and 6 from the enhanced active choice.

Our primary hypothesis, based on the findings of [8] is that actual attendance rates will be higher for enhanced active choice invitations than for active choice, which will in turn be higher than those for standard opt-in invitations.

Secondly, since previous work has found that making active choices can lead to more commitment to the decision made [11], we hypothesize that both the active choice and the enhanced active choice treatments will have less drop-off from saying they will attend to actual attendance, and from attending one day to attending the full five days.

### Estimation

To test the impact of treatment type on attendance, we estimate simple ordinary least squares regressions of the following form:
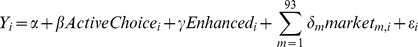



Where Y_i_ is the attendance outcome of interest, *ActiveChoice_i_* and *Enhanced_i_* are dummy variables indicating assignment to the active choice and enhanced active choice invitation types respectively, and *market_i,m_* is a dummy variable taking the value one if individual *i* is in market *m*, in order to account for randomization within market strata[15]. Huber-White standard errors are then used. The coefficients β and γ then give the differential impact of the active choice and enhanced active choice invitations relative to the opt-in invitation type.

One potential reason for failure to see any differential effect of treatment invitation type would be if attendance rates are high, with the decision to not attend not being affected by the relative costs and benefits of attending, but only by idiosyncratic factors. To explore this, we estimate probit regressions of the likelihood of attending as a function of the individual and business characteristics of women in our sample.

## Results

Eighty-seven percent of those invited to training said they would attend, and 76.4 percent attended at least one day. 94.6 percent of those who attended at least one day continued to finish all five days of training.

**Figure 1:**
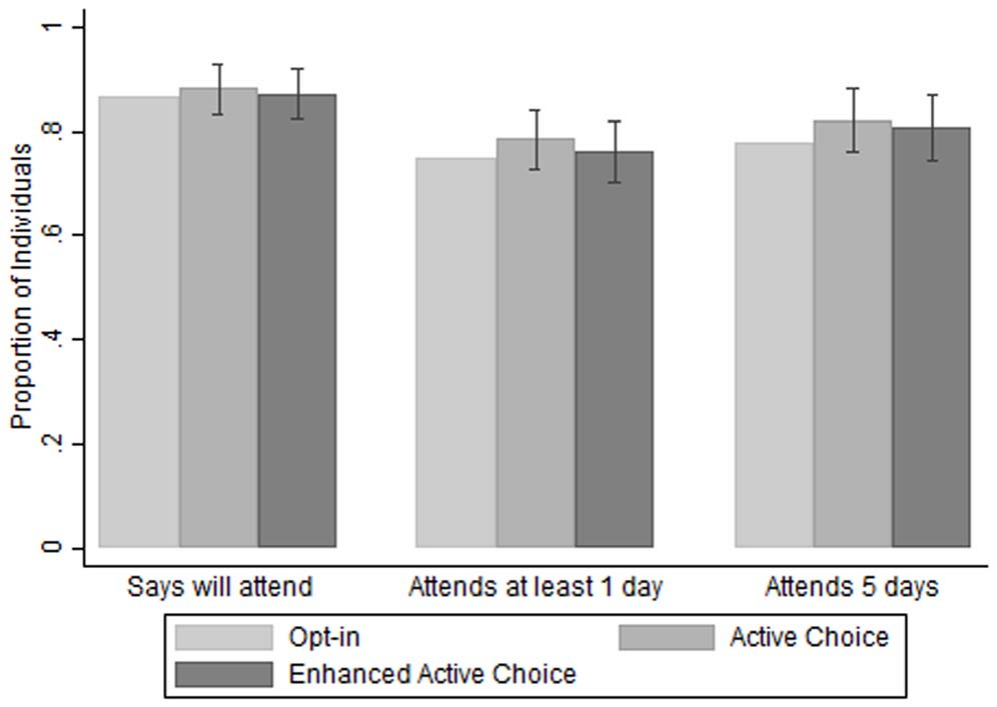
Impact of Invitation Type on Potential and Actual Business Training Attendance. Opt-in bars represent attendance rates for the opt-in invitation group. Bars for the active choice and enhanced active choice groups are the sum of the opt-in rate and the treatment coefficient estimated by OLS regression. OLS regression includes controls for marketplace. Lines on the active choice and enhanced active choice treatment bars represent plus or minus 1.96 times the standard error of the treatment coefficient. Attended all 5 days is conditional on attending at least one day.

Figure 1 shows similar rates across invitation treatment groups in all three measures of attendance. Table 3 shows the point estimates of the treatment effects are positive for both the active choice and enhanced active choice treatments, but small in magnitude and not statistically significant. Those receiving the active choice treatment were 3.8 percentage points more likely to attend training (p = 0.199) than the opt-in group, while those receiving the enhanced active choice were 1.8 percentage points more likely to attend (p = 0.556).

**Table 3:** Impact of Treatment Type on Attendance.

	Says willattend	Attends atleast 1 day	Attends all5 days	Attends all 5 daysafter saying will attend
Active Choice	0.017	0.038	0.041	0.032
	(0.024)	(0.029)	(0.031)	(0.030)
Enhanced Active Choice	0.008	0.018	0.031	0.026
	(0.024)	(0.030)	(0.032)	(0.031)
Mean for Opt-in Invitation	0.865	0.746	0.700	0.779
Sample Size	1172	1172	1172	1022
P-value for testing equality of treatments	0.759	0.437	0.397	0.535

Notes: Huber-White Standard error in Parentheses. *, **, and *** indicate significance at the 10, 5 and 1 percent levels respectively. Coefficients from OLS regressions after controlling for marketplace dummies.

The final column of Table 3 looks at whether individuals follow through on their initial agreement, by considering the fraction of those who say they will attend training who then follow on to attend all 5 days of training: done by 77.9 percent of the opt-in group. The rates of follow through are 2 to 3 percentage points higher for the active choice and enhanced active choice groups, and we can't reject equality of follow-through rates across all three groups (p = 0.535). Table S1 in File S1 shows that these results are robust to including additional controls such as participation in previous training courses, and variables that affect the probability of attendance in Table 4 below.

**Table 4:** Correlates of Training Attendance.

Dependent Variable: Attended at least one day of Training		
	(1)	(2)
Aged above 35	0.353***	0.368***
	(0.097)	(0.097)
Years of Schooling	0.021	0.018
	(0.017)	(0.017)
Married	−0.238**	−0.226**
	(0.102)	(0.104)
Has a child	0.087	0.113
	(0.177)	(0.179)
Has a child of 5 and under	−0.147	−0.142
	(0.096)	(0.096)
Household Size	0.110***	0.107***
	(0.029)	(0.030)
High discount rate	0.174**	0.194**
	(0.089)	(0.090)
Hyperbolic discounter	0.010	0.022
	(0.102)	(0.103)
Raven test score	0.016	0.020
	(0.019)	(0.019)
Digitspan recall	0.005	−0.003
	(0.044)	(0.045)
Owner has previously participated in business training	0.388**	0.420**
	(0.182)	(0.182)
Sector is retail	0.178	0.200*
	(0.113)	(0.114)
Keeps business records	0.123	0.119
	(0.113)	(0.113)
Has a non-family member employee	0.163	0.161
	(0.143)	(0.143)
Weekly profits (in 1000 s of KSH)	−0.111**	−0.116**
	(0.049)	(0.049)
Capital stock (excluding land and buildings) (1000 s of KSH)	0.001	0.001
	(0.001)	(0.001)
Total business practices score	0.011	0.011
	(0.010)	(0.010)
Distance to training above 10 km	−0.159*	−0.138
	(0.092)	(0.096)
Region dummies	No	Yes
Sample Size	1101	1101

Notes: Robust standard errors in parentheses. *, **, and *** indicate significance at the 10, 5 and 1 percent levels respectively. Coefficients shown are marginal effects from a probit regression.

Although invitation type has no impact on attendance, Table 4 shows that attendance rates do differ significantly along a number of other observable dimensions. Age and marital status are strong and statistically significant predictors of attendance: all else equal, women aged above 35 are 35 percentage points more likely to attend training than those below 35, while married women are 24 percentage points less likely to attend than unmarried women. This potentially reflects the competing demands on their time from other household tasks. Women are also more likely to attend if they have previously participated in training (perhaps reflecting greater perceived benefits from attending), have a large household (potentially providing more people to undertake household and business tasks in their absence), and are located closer to the training venue (reducing travel time). Women who earn more profits are less likely to attend, perhaps reflecting a higher opportunity cost of time, or that they think there is less need to improve. The one statistically significant variable that goes in the opposite direction to our priors is having a high discount rate, which is associated with greater attendance. This variable has a much smaller coefficient (0.036) and is not statistically significant (p = 0.148) when we enter that variable by itself in the probit regression, suggesting part of the effect is due to offsetting correlations with other variables. Taken together this table suggests that there is sizeable variation among women in our sample in their likelihood of attending in a way that corresponds to some of the costs and perceived benefits they face in attending training.

We also note that qualitative reports from our field team were that the main two reasons given by women for not attending were inability to find childcare, and not having someone to look after the business in their absence. Yet Table 4 shows that neither is a significant predictor of attendance. This suggests women are giving what they think are socially desirable answers when justifying their decision not to attend, but as Table 4 indicates, in practice they appear to be basing this decision at least in part on the perceived costs and benefits of attending.

## Discussion

Despite the promise offered by the active choice and enhanced active choice approaches, we find no significant effect on take-up in a business training program of using either approach compared to a more standard opt-in approach. This leads to the question of why do we fail to detect an effect?

A first possible explanation is that we fail to detect an impact of these alternative invitation types because of a lack of statistical power. Our sample size of almost 400 in each treatment group is relatively large by the context of many developing country field experiments: the mean (median) of 16 business training experiments summarized in [1] has 213 (151) individuals invited to training. The sample size also greatly exceeds the total samples of 55 and 110 used in the lab experiments of [8]. However, it is lower than the 2000 or more individuals in each treatment arm of [9] and the field experiments of [8]. Power calculations give that we have 80 percent power to detect an 8.5 percentage point increase in attendance on an assumed base of 75 percent. This is lower than the effect sizes of 9.6 percent and 16.3 percent found in [8], and 28 percent found in [9]. So we do have sufficient power to detect effect sizes of the magnitude found in other studies. Indeed, the 95 percent confidence interval for the active treatment effect on attendance is [−1.9%, +9.5%], while for the enhanced active choice treatment it is [−4.1%, +7.7%]. These are relatively narrow bounds, and suggest that it is not lack of statistical power that prevents us from detecting sizeable impacts of these choice structures.

A second potential explanation could be that we are testing these policies in a context of high attendance, in which everyone wants to attend and there is little room to affect decisions. But our probit results show that there are considerable differences in attendance rates across observable dimensions in a way that suggests people are, at least in part, weighing potential costs and benefits of attending. Since age is a strong predictor of attendance, the first two columns of Table 5 examine the treatment effects separately for those above and below the median age of 35. While the point estimates suggest slightly higher impacts of the treatment for the younger group, who have lower attendance rates, the difference in effect size is not large in magnitude and is not statistically significant.

**Table 5:** Impact of Treatment Type on Attendance for Different Subgroups.

Dependent Variable: Attended at least one day of training									
	Aged> 35	Aged< = 35	HighDecisionPower	MediumDecisionPower	LowDecisionPower	12+ years ofschooling	<12 yearsschooling	AbovemedianRaven	BelowmedianRaven
Active Choice	0.033	0.066	0.093*	0.051	−0.034	0.055	0.033	0.066	0.023
	(0.037)	(0.047)	(0.055)	(0.062)	(0.065)	(0.064)	(0.035)	(0.051)	(0.037)
Enhanced Active Choice	0.001	0.049	0.032	0.013	0.013	0.087	−0.008	0.087	−0.001
	(0.039)	(0.047)	(0.054)	(0.063)	(0.064)	(0.066)	(0.037)	(0.054)	(0.039)
Opt-in Mean	0.841	0.657	0.752	0.748	0.733	0.745	0.746	0.723	0.759
Sample Size	567	605	404	391	374	319	853	480	692
P-value for testing that:									
Active choice equal		0.585			0.413		0.908		0.432
Enhanced active choice equal		0.48			0.748		0.210		0.305

Notes: Huber-White Standard error in Parentheses. *, **, and *** indicate significance at the 10, 5 and 1 percent levels respectively.

Coefficients are from OLS regressions after controlling for marketplace dummies.

P-values are for test of equality of treatment effect across subgroups.

A third potential explanation suggested by a reviewer is that the cost of saying no may be perceived as lower in our context than was the case in the flu shot application of [8]. We note that KSH 17,000 is equivalent to 15 weeks of income for these women, which likely far exceeds the income loss that would be experienced from contracting influenza. The cost of not attending also seems likely to be higher than the cost of not automatically refilling prescriptions in [8]. Nevertheless, we acknowledge it is difficult to compare directly the size of the loss associated with the negative option in our context to that in previous health applications. This provides a further rationale for testing the effectiveness of choice structure in our context.

A fourth potential explanation is that the problem might be that the women being invited to training are not in fact the main decision-making agents in the decision of whether or not they attend. If household decision-making is ultimately made by the woman's spouse or parent, then the invitation type may instead need to be directed at the person in charge of making the decision of whether or not to attend. To examine this possibility, we use a question on the baseline survey which asked women whether they needed someone else's permission to travel to another location for work. We classify them as having high decision-making power if they said they did not (34%), medium decision-making power if they said they needed to inform another household member, but didn't need explicit permission (33%), and as having low decision-making power if they needed to request permission from another household member first (32%).

Columns 3 to 5 of Table 5 show that the active choice treatment has a 9.3 percentage point increase on attendance relative to opt-in for women with high decision-making power, with this significant at the 10 percent level. In contrast, the point estimate is lower (5.1 percentage points) for medium decision-making power, and negative and statistically insignificant (−3.4 percentage points) for low decision-making power. This is consistent with the active choice method being more effective when the person it is directed to is the main decision-maker, but we cannot reject equality of treatment effects for the three decision-making levels (p = 0.413 for active choice).

A fifth possibility is that the active choice, and especially enhanced active choice, language is too complicated for poor, relatively uneducated women. To test this we examine heterogeneity in treatment impact with respect to whether or not they have at least 12 years of schooling (25% do), and to whether or not they score above the median score on a Raven Progressive Matrix test (a measure of abstract reasoning, often considered a general intelligence test). The last columns of Table 5 show point estimates consistent with this hypothesis, with the enhanced active choice treatments have a 9 percentage point higher effect for individuals with more education or higher ability, and the active choice treatments 2 to 3 percentage point higher effects. Nevertheless, we cannot reject equality of treatment effects for these subgroups.

These results are therefore suggestive of the idea that requiring an active decision has more effect when this is required of the person with the power to make this decision, and that the enhanced active choice approach is more effective when delivered to people with higher reasoning levels. However, while our study has sufficient statistical power to rule out large overall effects, power is much lower and confidence intervals much wider once we start looking at these subgroups. It is therefore of interest for future work to further examine the applicability of active choice and enhanced active choice methods to developing country settings in which education levels are lower and decision-making power more dispersed than in the developed country settings for which these methods were developed.

## Supporting Information

File S1 (PDF)
