# Supplementary material for: Invitation Choice Structure Has No Impact on Attendance in a Female Business Training Program in Kenya
Source: PLoS One. 2014 Oct 9;9(10):e109873. doi: 10.1371/journal.pone.0109873 (PMC4192542; doi:10.1371/journal.pone.0109873)
Supplement: File S1 — (PDF) [file pone.0109873.s001.pdf]

Invitation choice structure has no impact on attendance in a female business training program in Kenya

FILE S1: SUPPLEMENTARY MATERIALS

Example of Training Invitation (English Version)

All three invitation types received the same information about the training:

The ILO Women Entrepreneurship Development and Economic Empowerment (WEDEE) Programme in partnership with Kenya Industrial Estates (KIE)- has organized a 5 days' workshop on business management skills called *GET Ahead*.

The training will take place next week, from Monday the 24th to Friday the 28th of June 2013 from 8:30AM to 4:30PM. The training will be held at the Kakamega Catholic Church Hall. Kakamega Catholic Church is located opposite Somken Fuel Station, or diagonally opposite Masinde Muliro Gardens in Kakamega.

The objective of this workshop is to train you and build your capacity to operate your business or enable you to set up and operate a new business efficiently. This training typically costs 17,000 KSH to provide, and even though it is usually subsidized, most NGOs charge at least 2,000 KSH for it. **We are pleased to offer it to you for free.**

ILO shall cater for the following;

- Travel allowance for 5 days
- Meals and refreshments during training
- Training manuals
- Certificate of attendance

The above allowance shall be provided based on full participation in all 5 days, and upon signing the daily participant's register.

The scripts then differed in how the choice to attend was presented.

[Opt-in Invitation]

You have been selected from the survey that is being carried out and invited to participate in this training workshop. *Please let me know if:*

- *<u>you will participate</u> in the GET AHEAD training program*

[**Active Choice Invitation]**

You have been selected from the survey that is being carried out and invited to participate in this training workshop. *Please let me know if (**READ BOTH OPTIONS OUT LOUD AND MARK THE CHOICE OF THE INVITEE ON THE EXCEL LIST**):*

- *<u>you will participate</u> in the GET AHEAD training program*
- *<u>you will not participate</u> in the GET AHEAD training program*

[Enhanced Active Choice Invitation]

You have been selected from the survey that is being carried out and invited to participate in this training workshop. *Please let me know if (**READ BOTH OPTIONS OUT LOUD AND MARK THE CHOICE OF THE INVITEE ON THE EXCEL LIST**):*

- *you will participate in the GET AHEAD training program in order to learn new business skills that could help you grow your business and take advantage of free training valued at KSH 17,000.*
- *you will not participate in the GET AHEAD training program, and will turn down the opportunity to learn new skills for your business and choose not to receive KSH 17,000 of free training.*

Example of Training Invitation (Swahili Version)
All three invitation types received the same information about the training:

Shirika la ILO Women Entrepreneurship Development and Economic Empowerment (WEDEE) Programme, wakishirikiana na Kenya Industrial Estates (KIE)-wameandaa warsha ya siku 5 kuhusu maarifa katika usimamizi wa biashara yanayoitwa GET Ahead.

Mafunzo yatafanyika wiki ijayo, kuanzia Jumatatu tarehe 24 hadi Ijumaa, tarehe 28 Juni 2013 kuanzia 8.30 asubuhi hadi 4.30 jioni. Mafunzo haya yatafanyika katika ukumbi wa kanisa la Catholic Kakamega. Kanisa la Catholic la Kakamega linaangaliana na Somken Petrol Station au Masinde Muliro Gardens, mjini Kakamega.

Lengo la warsha hii ni kukufunza na kukupa uwezo wa kuendesha biashara yako au kukuwezesha kuanzisha na kuendesha biashara mpya kwa ufanisi. Kwa kawaida, mafunzo haya hugharimu Ksh. 17,000 kuandaa, na hata kama hii kwa kawaida hupunguzwa, mashirika mengi yasiyo ya kiserikali hulipisha Ksh. 2,000 kwa haya. **Tunafuraha ya kukupa haya bila malipo.**
ILO itasimamia yafuatayo;

- Nauli ya usafiri kwa siku 5
- Vyakula na vinywaji wakati wa mafunzo
- Miongozo (manuals) ya mafunzo
- Cheti cha kuhudhuria

Nauli iliyotajwa itapeanwa kulingana na kushiriki kikamilifu kwa siku zote 5, na kwa kutia sahihi katika kitabu cha kujiandikisha cha kila siku.

The scripts then differed in how the choice to attend was presented.

[Opt-in Invitation]
Umechaguliwa kutokana na utafiti unaoendelea na umealikwa kushiriki katika warsha hii ya mafunzo. *Tafadhali nieleze ikiwa:*

- *Utashiriki katika mafunzo ya mpango wa GET AHEAD*

[**Active Choice Invitation]**
Umechaguliwa kutokana na utafiti unaoendelea na umealikwa kushiriki katika warsha hii ya mafunzo. *Tafadhali nieleze ikiwa: (**READ BOTH OPTIONS OUT LOUD AND MARK THE CHOICE OF THE INVITEE ON THE EXCEL LIST**):*

- *Utashiriki katika mafunzo ya mpango wa GET AHEAD*
- *Hutashiriki katika mafunzo ya mpango wa GET AHEAD*

[Enhanced Active Choice Invitation]
Umechaguliwa kutokana na utafiti unaoendelea na umealikwa kushiriki katika warsha hii ya mafunzo. *Tafadhali nieleze ikiwa: (**READ BOTH OPTIONS OUT LOUD AND MARK THE CHOICE OF THE INVITEE ON THE EXCEL LIST**):*

- *Utashiriki katika mafunzo ya mpango wa GET AHEAD ilikujifunza maarifa mapya ya biashara yanayoweza kukusaidia kukuza biashara yako na kupata manufaa ya mafunzo ya bure yenye thamani ya Ksh. 17,000.*
- *Hutashiriki katika mafunzo ya mpango wa GET AHEAD, na kupuuza fursa ya kujifunza maarifa mapya kwa biashara yako na kuchagua kutopokea mafunzo ya bure yenye thamani ya Ksh. 17,000*

Appendix Table S1 shows that the results in Table 3 are robust to inclusion of other controls.

Appendix Table S1: Impact of Treatment Type on Attendance - Robustness Checks

	Says will attend	Attends at least 1 day	Attends all 5 days	Attends all 5 days after saying will attend
Panel A: Coefficients as in Table 3 of the paper				
Active Choice	0.017	0.038	0.041	0.032
	(0.024)	(0.029)	(0.031)	(0.030)
Enhanced Active Choice	0.008	0.018	0.031	0.026
	(0.024)	(0.030)	(0.032)	(0.031)
Panel B: controlling for previous training attendance				
Active Choice	0.015	0.033	0.036	0.028
	(0.024)	(0.029)	(0.031)	(0.030)
Enhanced Active Choice	0.008	0.019	0.032	0.028
	(0.024)	(0.030)	(0.032)	(0.031)
Panel C: controlling for variables that are significant predictors of attendance in Table 4				
Active Choice	0.024	0.037	0.040	0.030
	(0.024)	(0.029)	(0.031)	(0.030)
Enhanced Active Choice	0.005	0.013	0.026	0.021
	(0.024)	(0.030)	(0.031)	(0.030)

Notes: panel C controls for age above 35, being married, retail, profits, being more than 10km from the training location, previously attending any training, household size, and discount rate
